# Water molecules bonded to the carboxylate groups at the inorganic–organic interface of an inorganic nanocrystal coated with alkanoate ligands

**DOI:** 10.1093/nsr/nwab138

**Published:** 2021-08-04

**Authors:** Jiongzhao Li, Weicheng Cao, Yufei Shu, Haibing Zhang, Xudong Qian, Xueqian Kong, Linjun Wang, Xiaogang Peng

**Affiliations:** Zhejiang Key Laboratory of Excited-State Materials, Department of Chemistry, Zhejiang University, Hangzhou 310027, China; Zhejiang Key Laboratory of Excited-State Materials, Department of Chemistry, Zhejiang University, Hangzhou 310027, China; Zhejiang Key Laboratory of Excited-State Materials, Department of Chemistry, Zhejiang University, Hangzhou 310027, China; Zhejiang Key Laboratory of Excited-State Materials, Department of Chemistry, Zhejiang University, Hangzhou 310027, China; Zhejiang Key Laboratory of Excited-State Materials, Department of Chemistry, Zhejiang University, Hangzhou 310027, China; Zhejiang Key Laboratory of Excited-State Materials, Department of Chemistry, Zhejiang University, Hangzhou 310027, China; Zhejiang Key Laboratory of Excited-State Materials, Department of Chemistry, Zhejiang University, Hangzhou 310027, China; Zhejiang Key Laboratory of Excited-State Materials, Department of Chemistry, Zhejiang University, Hangzhou 310027, China

**Keywords:** nanocrystals, quantum dots, water, carboxylate ligands, interface

## Abstract

High-quality colloidal nanocrystals are commonly synthesized in hydrocarbon solvents with alkanoates as the most common organic ligand. Water molecules with an approximately equal number of surface alkanoate ligands are identified at the inorganic–organic interface for all types of colloidal nanocrystals studied, and investigated quantitatively using CdSe nanocrystals as the model system. Carboxylate ligands are coordinated to the surface metal ions and the first monolayer of water molecules is found to bond to the carboxylate groups of alkanoate ligands through hydrogen bonds. Additional monolayer(s) of water molecules can further be adsorbed through hydrogen bonds to the first monolayer of water molecules. The nearly ideal environment for hydrogen bonding at the inorganic–organic interface of alkanoate-coated nanocrystals helps to rapidly and stably enrich the interface-bonded water molecules, most of which are difficult to remove through vacuum treatment, thermal annealing and chemical drying. The water-enriched structure of the inorganic–organic interface of high-quality colloidal nanocrystals must be taken into account in order to understand the synthesis, processing and properties of these novel materials.

## Teaser

Coupled with computational means, FTIR and nuclear magnetic resonance spectroscopy measurements identify that water molecules are predominately bonded to the carboxylate groups of alkanoate ligands via hydrogen bonds.

## INTRODUCTION

Colloidal inorganic nanocrystals, including semiconductor nanocrystals with sizes in the quantum-confinement regime (quantum dots, QDs), are nanometer-sized fragments of the corresponding bulk single crystal stabilized with a monolayer of organic ligands [1]. Surface ligands affect nearly all aspects of colloidal nanocrystals, such as function, solution processibility and synthesis [2]. For instance, colloidal QDs are widely explored in high-performance display technologies [3], bio-medical diagnostics [4,5], photocatalysis [6,7] and photovoltaic devices [8–10], each of which requires specifically tailored ligands. Effects of the ligand monolayer on the synthetic chemistry of high-quality colloidal nanocrystals are well known, especially those performed in non-aqueous solvents [11–22]. The solubility of colloidal nanocrystals can be boosted by 2–6 orders of magnitude by introducing entropic ligands [18]; this is needed for the fabrication of solution-processed optoelectronic devices.

The molecular picture of the ligand monolayer on a colloidal nanocrystal is ambiguous, especially at the interface between the inorganic nanocrystal and its organic ligands (inorganic–organic interface). High-quality nanocrystals synthesized in non-aqueous solutions are usually single crystalline in nature, and thus the interior inorganic structure can be well characterized using diffraction techniques and transmission electron microscopy [23,24]. In most cases, the composition and molecular structure of organic ligands are not too difficult to clarify [25–27]. Since 2001 [12], fatty acids and their metal salts have become the most common ligands for synthesis of high-quality nanocrystals, with their carboxylate groups bonded onto the surface metal sites of a nanocrystal. With sufficient configurational entropy from their flexible hydrocarbon chains, the alkanoate monolayer on a nanocrystal provides a hydrophobic outer surface region to ensure desirable solution dispersity in non-polar solvents for both synthesis and processing [18,28].

At the inorganic–organic interface, the situation is rather complex. It is evidently a transition zone with multi-functions i.e. from randomly packed and flexible organic phase to crystalline inorganic phase, from covalently bonded hydrocarbon chains to nearly ionic inorganic crystals, and from a hydrophobic and non-polar region to a strongly polar and hydrophilic region. For the most common ligand systems, i.e. fatty acid/fatty acid salts, chemical bonding between the surface metal sites and carboxylate groups has been extensively studied experimentally and theoretically [27,29,30]. As for the hydrophilic environment around the interface, there is not much knowledge.

Theoretical analysis suggested the existence of negatively charged OH^–^ groups at the interface for PbS QDs coated with alkanoate ligands, though the measurements were brief [30]. It was identified that, at the interface of InP QDs, there might be various forms of indium oxide species [17,31]. Synthesis of different types of high-quality oxide nanocrystals was found to be possible by simply heating up the corresponding metal fatty acid salts in hydrocarbon solvents, such as octadecene [13,14,32]. After dehydrogenation of octadecene by sodium metal, hydrolysis of indium carboxylates with trace amounts of residual water molecules occurred readily to form high-quality In_2_O_3_ nanocrystals [32], implying a strong capability of enriching water at the interface for continuous growth of the nanocrystals. It was suspected that there might be some water-related variation of emission properties of CdSe/CdS core/shell QDs [33,34].

Based on the facts discussed in the above paragraph, we hypothesize that there should be some water molecules adsorbed at the inorganic–organic interface for inorganic nanocrystals with carboxylate ligands. Because the commonly applied characterization methods for colloidal nanocrystals are insensitive to water and contamination is generally not carefully avoided, such water molecules have yet to be studied. Here, coupled with chemical means and computational efforts, Fourier transform infrared (FTIR) spectroscopy and nuclear magnetic resonance (NMR) spectroscopy methods with isotope labeling are developed to circumvent the hurdles of characterization. Results reveal that, on average, there is ∼1 monolayer of water molecules at the inorganic–organic interface of different types of nanocrystals coated with alkanoate ligands, which are mostly bonded to the carboxylate groups with hydrogen bonds, instead of directly onto the inorganic surface sites.

## RESULTS AND DISCUSSION

### CdSe QDs as the model system

**Figure 1.**
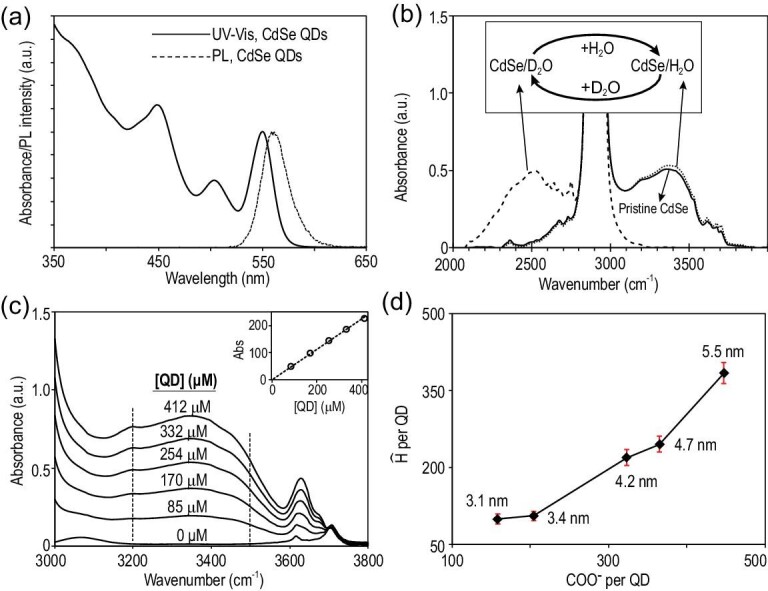
(a) UV–Vis and photoluminescence (PL) spectra of CdSe QDs with alkanoate ligands. (b) FTIR spectra of CdSe QDs with water and deuterium oxide treatments. ‘Pristine CdSe’ refers to the CdSe QDs before any H_2_O/D_2_O treatments. (c) FTIR spectra of CdSe QDs with concentration range between 0 μM and 412 μM. Inset: integrated FTIR absorbance between 3200 cm^–1^ and 3500 cm^–1^ versus the QD concentration. (d) Number of hydrogen atoms in hydroxy groups verses number of carboxylates per CdSe QD with different sizes.

Nearly monodisperse CdSe QDs in zinc-blende structure with alkanoate ligands are applied as the main model system, and are synthesized in octadecene, with cadmium alkanoates (with excess fatty acids) and selenium powder as the precursors (see Experimental Section in supporting information for detail) [35]. Their size-dependent optical properties (see Fig. 1a, for example) can be applied as convenient probes for determining size, size distribution and concentration of the nanocrystals. Specifically, the average size is determined using the absorption peak, its sharpness offers information about their size distribution, and the QD concentration is determined using their known extinction coefficients [36]. Though CdSe QDs with different sizes are studied, results below shall be based on the QDs with a diameter of 3 nm and their first excitonic absorption peak at 550 nm (Fig. 1a) unless otherwise stated.

CdSe QDs are purified thoroughly using a literature procedure with slight modifications [36]. Briefly, the as-synthesized QDs with stearate ligands are reacted with capric acids to offer excellent dispersity in any non-polar solvents at room temperature. Multiple cycles of dissolution/precipitation are carried out to remove any unreacted metal carboxylates and free fatty acids. Previous studies identified bonding configurations of carboxylates to the surface cadmium sites [27], and therefore offer a needed reference for understanding incorporation of water molecules at the interface.

### O–H vibration from the molecules at the inorganic–organic interface of CdSe QDs

Figure 1b illustrates FTIR spectra of the purified CdSe QDs dissolved in CCl_4_ (labeled as Pristine CdSe in Fig. 1b), and shows strong yet broad infrared (IR) absorption between 3000–4000 cm^–1^. In comparison, the trace amount of water in the solvent (CCl_4_) shows two sharp vibration peaks for free water at 3630 cm^–1^ (symmetric) and 3710 cm^–1^ (asymmetric) in the spectral range (Fig. S1, Supplementary Data). In addition to two sharp peaks with relatively low absorbance, the very broad and strong peak between 3200–3580 cm^–1^ should be associated with bonded water and/or other species with O–H vibration motif, such as neutral methanol molecules and the negatively charged hydroxyl groups (OH^–^) bonded onto the surface Pb sites for PbS QDs [30]. To verify this assignment, Pristine CdSe QDs are allowed to react with a large excess of deuterium oxide (D_2_O) added on top of the CCl_4_ solution as a separate phase at room temperature. Under such conditions, it is well known that active hydrogens in the form of O–H groups can readily go through hydrogen–deuterium exchange. After removal of the D_2_O layer, FTIR measurements of the exchanged QDs (labeled as ‘CdSe/D_2_O’ in Fig. 1b) in the CCl_4_ phase reveal that the entire broad and strong IR band above 3000 cm^–1^ disappears while the C–H vibration band of the ligands between ∼2800–3000 cm^–1^ remains. Simultaneously, a broad and strong IR band appears between 2200–2800 cm^–1^ for the exchanged QDs, in good accordance with O–D vibrations (Fig. S1, Supplementary Data). Figure 1b further shows that this H–D exchange process is fully reversible by reacting CdSe/D_2_O with H_2_O (the recovered QDs being labeled as ‘CdSe/H_2_O’ in Fig. 1b).

Figure 1c demonstrates a series of FTIR spectra of the CdSe QDs with different QD concentrations in CCl_4_. While absorbance of the sharp IR peak at 3710 cm^–1^ associated with free water in the solution remains constant, absorbance of the broad IR band and some of the sharp IR peaks all increase upon increasing the QD concentration in the solution. We choose the integrated absorbance between 3200–3500 cm^–1^ as the quantitative measure for the O–H vibrations of the interface-bonded species, which avoids possible overlapping with the C–H vibrations below ∼2800–3000 cm^–1^ and residual sharp IR peaks above 3500 cm^–1^ from the free water molecules in the solution. For convenience, the O–H vibration motif shall be denoted as ‘active hydrogen atom (${\rm{\hat{H}}}$)’ for short, referring to those that can be efficiently exchanged with deuterium atoms.

As shown in the inset of Fig. 1c, the integrated absorbance between 3200–3500 cm^–1^ is strictly proportional to the QD concentration in the solution, implying direct association of the broad IR band with the QDs. This conclusion is further supported by Fig. 1d, which reveals that the number of active hydrogen atoms (${\rm{\hat{H}}}$) per QD increases monotonically as the size of CdSe QDs increases. Methods for quantifying the absolute number of ${\rm{\hat{H}}}$ per QD shall be introduced below.

### Nature of the O–H motif

**Figure 2.**
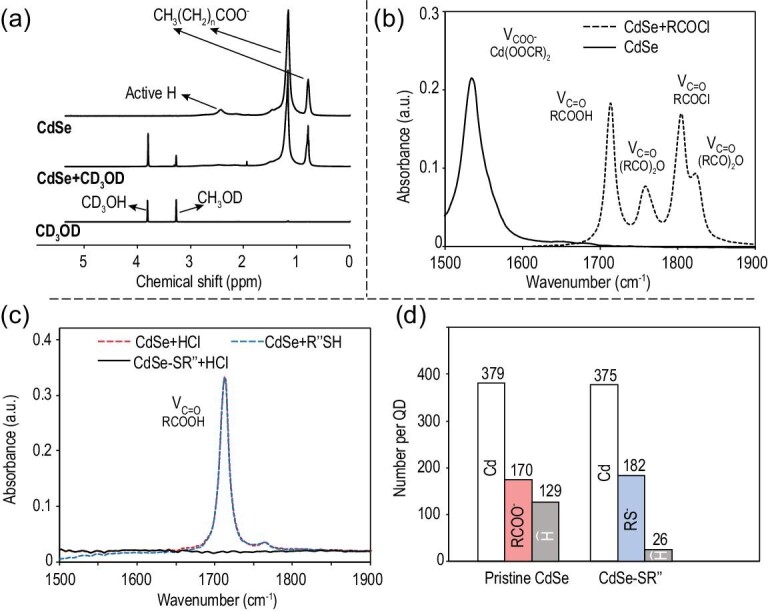
(a) ^1^H NMR spectra of CD_3_OD, CdSe QDs and CdSe QDs mixed with CD_3_OD in CDCl_3_. (b) FTIR spectra of CdSe QDs with/without the acyl-chloride treatment. (c) FTIR spectra of CdSe QDs reacted with HCl, reacted with thiol and reacted with HCl after the thiol treatment (with additional purification). (d) Number of atoms/molecules per QD for Pristine CdSe and thiol-coated CdSe QDs (CdSe-SR’’).

NMR measurements (Fig. 2a) reveal that, in addition to the broad ^1^H NMR signals of alkanoate ligands, a broad ^1^H NMR signal appears at ∼2.57 ppm for the QDs in CDCl_3_, which is consistent with the active hydrogen atoms in the form of interface-bonded molecules, either water or the negatively charged OH^–^ group. Upon addition of deuterium methanol (CD_3_OD) into the CDCl_3_ solution of the QDs, the broad ^1^H NMR signal at ∼2.57 ppm disappears. Instead, relative to the signal of hydroxyl-deuterated methanol (CH_3_OD), the hydroxyl hydrogen peak of the methyl-deuterated methanol (CD_3_OH) increases its intensity by ∼3 times. These results confirm that there are hydroxyl species on the QD surface, which can be efficiently converted to deuterium oxide species through the hydrogen–deuterium exchange with added CD_3_OD. Furthermore, the active hydrogen atoms (or surface hydroxyl groups) are not in the form of interface-bonded methanol, though methanol is applied for purification of the QDs.

Liquid-phase FTIR measurements (Fig. 2b) show that there is no free fatty acid in the solution of the purified QDs, excluding another possible form of the surface hydroxyl groups. Into this solution of QDs in dodecane, an excess amount of acryl chloride (RCOCl) is added. This completely removes the IR peak at ∼1537 cm^–1^ for asymmetric vibration of the carboxylate groups, and a few new peaks appear in the region for other types of carbonyl species in the solution, including free carboxylic acid (RCOOH), acid anhydride ((RCOO)_2_O) and excess acryl chloride (Fig. 2b). Control experiments confirm that, without the purified CdSe QDs in the solution, there is no formation of either free fatty acids or acid anhydrides by addition of acryl chlorides (Fig. S2, Supplementary Data). For the current system, these products should be produced through the following reactions.

(1) \begin{eqnarray*} &&{\rm{QD}} {-} {\left( {{\rm{Cd}}{{\left( {{\rm{OOCR}}} \right)}_x}} \right)_n} + {\rm{RCOCl}} \to\nonumber\\ &&\qquad{\rm{QD}} {-} {\left( {{\rm{Cd}}{{\left( {{\rm{Cl}}} \right)}_x}} \right)_n} + {\left( {{\rm{RCOO}}} \right)_2}{\rm{O}} \end{eqnarray*}

\begin{eqnarray*} &&{\left( {{{\rm{H}}_2}{\rm{O}}} \right)_m} \hbox{-} {\rm{QD}} + {\rm{RCOCl}}\nonumber\\ &&\qquad \to {\left( {{\rm{HCl}}} \right)_m} \hbox{-} {\rm{QD}} + {\rm{RCOOH}} \end{eqnarray*}

QD-(Cd(OOCR^′^)*_x_*)*_n_* represents a CdSe QD with alkanoate ligands (total number of alkanoates per QD being *nx*), which reflects the fact that the carboxylate ligands are bonded with surface cadmium sites in different forms, with *x* = 0.5, 1 and 2 for (111), (100) and (110) (or other non-polar) facets, respectively [27]. Reaction between these carboxylate ligands and acryl chlorides would result in acid anhydrides (Equation (1)). After excluding the presence of methanol and carboxylic acid on the purified QDs, there are two possible reactions of acryl chlorides that yield free fatty acids from the purified QDs. Equation (2) illustrates the reaction between acryl chlorides and water molecules adsorbed on the QDs ((H_2_O)*_m_*-QD, total water molecules per QD being *m*). The other possible reaction would occur between acryl chloride and the negatively charged hydroxyl groups (OH^–^) bonded directly to the surface cadmium sites, which shall be excluded below. Figure 2b reveals that concentrations of the resulting fatty acids and acid anhydrides are similar to each other, given IR extinction coefficients of both types of carbonyl groups being similarly high [37].

**Figure 3.**
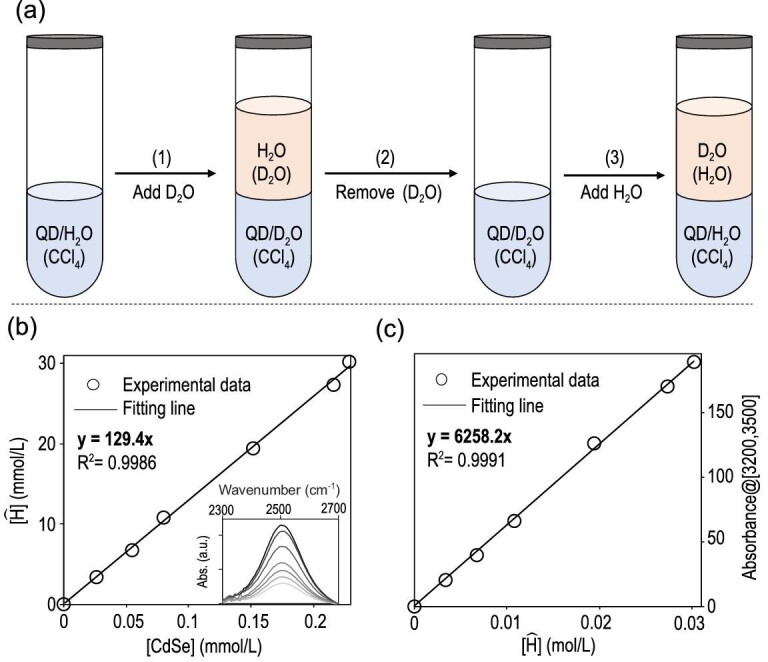
(a) Scheme of the hydrogen–deuterium exchange process. (b) Concentration of active hydrogen verses concentration of the QDs. Inset: FTIR spectra of D_2_O in H_2_O (D_2_O(H_2_O)) obtained by Step 3 in (a). (c) IR absorbance between 3200 cm^–1^ and 3500 cm^–1^ in the IR spectrum of a given sample verses concentration of active hydrogen atoms in the sample.

By reacting the purified CdSe QDs (Pristine CdSe) with excess HCl, all surface carboxylate ligands are converted to free fatty acids. In comparison, with the same concentration of the purified QDs, reaction between dodecanethiol (RSH) and the QDs results in an IR peak for free fatty acids with identical spectral shape and intensity (Fig. 2c). The resulting CdSe QDs coated with thiolate ligands (CdSe-SR) are purified and further reacted with HCl, which shows no IR signals in the carbonyl region (1500–1900 cm^–1^), indicating complete replacement of the carboxylate ligands by the thiolate ones (−SR). For Pristine CdSe and CdSe-SR, the carboxylate and thiolate ligands per QD are quantified (Figs S3 and S4, Supplementary Data). The results are plotted with cadmium atoms per QD as the reference (Fig. 2d). Figure 2d also includes interface-bonded water molecules per QD for each type of QD, determined using the method in Fig. 3a (see detail in the next subsection).

Evidently, replacement of surface ligands does not affect the total number of cadmium atoms per QD, which implies a one-on-one replacement of carboxylate ligands by thiolate ligands with the same negative charge (−1). As a control experiment, thiols are confirmed to readily react with Cd(OH)_2_ (Fig. S5, Supplementary Data). If there are any negatively charged hydroxyl groups bonded onto the surface cadmium sites, it should be in the form of Cd(OH)_x_, and the number of thiolate ligands per CdSe-SR QD should be equal to the sum of the carboxylate and OH^–^ surface groups. Figure 2d illustrates that the number of carboxylate ligands per Pristine CdSe is equivalent to a closely packed monolayer of the ligands [36] and only ∼7% less than that of the thiolate ligands per CdSe-SR QD. Because this difference is close to the overall experimental errors (3%–5% for each set of measurements) for the experiments (Fig. S4, Supplementary Data), it is reasonable to state that there are barely any negatively charged OH^–^ groups bonded on the surface of Pristine CdSe QDs. Thus, as concluded in Equation (2), the large amount of free fatty acids formed by the reaction between the Pristine CdSe QDs and acryl chlorides should solely result from the interface-bonded water molecules.

Furthermore, the results in Fig. 2c and d can be summarized by the following reactions (Equations (3) and (4)). In these reactions, both alkanoate ligands and adsorbed water molecules are presented, giving a formula for a QD as QD-(Cd(OOCR)*_x_*)*_n_*-(H_2_O)*_m_*. For the QDs with a specific size, it should be possible to calculate the stoichiometric coefficients *m*, *x* and *n* using the data in Figs 1d and 2d.

(3) \begin{eqnarray*} &&{\rm{QD}} \hbox{-} {\left( {{\rm{Cd}}{{\left( {{\rm{OOCR}}} \right)}_x}} \right)_n} \hbox{-} {\left( {{{\rm{H}}_2}{\rm{O}}} \right)_m} + {\rm{HCl}}\nonumber\\ &&\!\!\qquad \to {\rm{CdC}}{{\rm{l}}_2} + {{\rm{H}}_2}{\rm{Se}} + {{\rm{H}}_2}{\rm{O}} + {\rm{RCOOH}}\nonumber\\ \end{eqnarray*}

(4) \begin{eqnarray*} &&{\rm{QD}} \hbox{-} {\left( {{\rm{Cd}}{{\left( {{\rm{OOCR}}} \right)}_x}} \right)_n} \hbox{-} {\left( {{{\rm{H}}_2}{\rm{O}}} \right)_m} + {\rm{RSH}} \nonumber\\ &&\qquad\!\!\to {\rm{QD}} \hbox{-} {\left( {{\rm{Cd}}{{\left( {{\rm{SR}}} \right)}_x}} \right)_n} \hbox{-} {\left( {{{\rm{H}}_2}{\rm{O}}} \right)_{0.2m}}\nonumber\\ &&\qquad\qquad\!\! +\, {{\rm{H}}_2}{\rm{O}} + {\rm{RCOOH}} \end{eqnarray*}

Interestingly, the number of water molecules decreases substantially (∼80%) by the ligand exchange with thiolates (Fig. 2d and Equation (4)), though the number of original alkanoate ligands equals that of thiolate ligands. This suggests that the water molecules are predominately associated with the alkanoate ligands. Considering the molecular structure of the surface alkanoate ligands, one would suspect that they are most likely to appear at the hydrophilic region offered by the carboxylate groups of alkanoate ligands. Though these water molecules are neutral and should not be replaced by the negatively charged thiolate ligands, they should leave the QDs when the carboxylate groups are removed by the ligand exchange. Presumably, the residual water molecules (∼20%) after the ligand exchange should be those directly bonded on the non-polar facets of the QDs, which is consistent with their low coverage of cadmium alkanoate ligands [27].

### Quantitative determination of water molecules at the interface of a QD

Quantitative determination of the concentration of active hydrogen atoms in a solution is established by a hydrogen–deuterium exchange procedure (Fig. 3a). In Step 1, the purified QDs in CCl_4_ are treated by a large excess of D_2_O (∼10^4^ in excess), which converts the surface bonded H_2_O to D_2_O completely (see FTIR results in Fig. 1b) through either hydrogen–deuterium exchange or H_2_O–D_2_O exchange. The D_2_O phase with the exchanged H_2_O (labeled as H_2_O(D_2_O)) is removed in Step 2. In Step 3, a large excess of H_2_O is added onto the CCl_4_ solution of the QDs with the interface-bonded D_2_O, which converts the interface-bonded D_2_O back to H_2_O completely (see Fig. 1b). Simultaneously, the newly replaced D_2_O molecules are transported into the top H_2_O phase (D_2_O(H_2_O)). Measuring the IR spectrum of the top water phase obtained in Step 3, one would observe a characteristic IR band centered at ∼2500 cm^−1^ for D_2_O dissolved in H_2_O (Fig. 3b, inset). Using the extinction coefficients of D_2_O in H_2_O measured separately (Fig. S6, Supplementary Data), one can then determine the concentration of active hydrogen atoms of the interface-bonded H_2_O for a specific sample [38,39].

For the typical CdSe QDs (3.0 nm in size), a series of FTIR spectra are obtained using the method outlined above for the CCl_4_ solutions with different concentrations of QDs. Figure 3b plots the concentration of active hydrogen atoms versus the QD concentration in a specific solution, with the corresponding FTIR spectra summarized in Fig. 3b (inset). Results in Fig. 3b yield an excellent linear function and the slope is the number of active hydrogen atoms per Pristine QD. Specifically, each QD bonds 130 active hydrogen atoms at its inorganic–organic interface on average, corresponding to 65 water molecules at the inorganic–organic interface per CdSe QD (3.0 nm in size).

Determination of the concentration of interface-bonded water further allows measurement of the extinction coefficient of the related IR band. After measuring a series of FTIR spectra of Pristine CdSe QDs with known concentrations (see Fig. 1c for example), the integrated absorbance between 3200 and 3500 cm^–1^ is plotted against the concentration of the active hydrogen atoms of the interface-bonded water, resulting in an excellent linear function, with the slope as the molar extinction coefficient of the active hydrogen atoms in the interface-bonded water molecules, namely, 6258 (cm L/mol) between 3200 and 3500 cm^–1^. This provides a convenient probe for determining the number of interface-bonded water molecules per nanocrystal.

### Responses of interface-bonded water molecules to environment change

**Figure 4.**
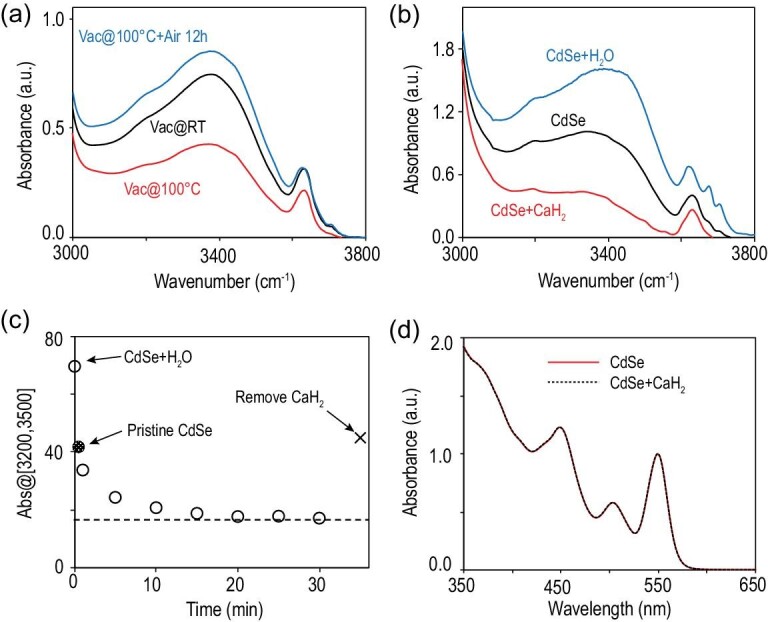
(a) FTIR spectra of CdSe QDs under vacuum at room temperature (Vac@RT), at 100^o^C for 4 hours (Vac@100^o^C) and re-exposed to air for 12 hours (Vac@100^o^C + Air 12h). (b) FTIR spectra of the freshly synthesized and purified CdSe QDs (fresh CdSe), and the QDs equilibrated with the water-saturated CCl_4_ solution (CdSe + H_2_O) and CaH_2_-treated (CdSe + CaH_2_). (c) Temporal evolution of absorbance between 3200 cm^–1^ and 3500 cm^–1^ during CaH_2_ treatment, with data points for two control experiments. (d) UV–Vis spectra of CdSe QDs before and after CaH_2_ treatment.

After purification, the QDs in powder form are placed in a vacuum oven. At room temperature, vacuum treatment can only reduce interface-bonded water molecules by ∼10% (Fig. 4a). With vacuum treatment at 100^o^C, the interface-bonded water molecules plateau at ∼50% of that of purified QDs (Fig. 4a). The samples after the vacuum treatments recover their water content effectively by exposure to air. These results reveal that the water molecules at the inorganic–organic interface are bonded quite strongly.

After equilibrium with the water phase on top of the CCl_4_ solution of QDs (see Fig. 3a for experimental set-up), absorbance of two sharp IR peaks for free water molecules in the CCl_4_ solution at 3630 and 3710 cm^–1^ increases significantly (Fig. 4b), consistent with saturation of water in CCl_4_. Simultaneously, the broad IR band related to the interface-bonded water molecules of the QDs in the CCl_4_ phase also increases its absorbance. In general, the number of interface-bonded water molecules on the freshly prepared CdSe QDs (Pristine CdSe QDs) varies somewhat while that of the QDs equilibrated with the water-saturated CCl_4_ is rather constant. After removal of the top water phase, the number of interface-bonded water molecules remains unchanged upon exposure to air for a long period of time. Usually, the number of interface-bonded water molecules on freshly prepared QDs is 60 ± 15% of that on the QDs equilibrated with the water-saturated CCl_4_.

Figure 4b shows that the addition of CaH_2_ powder into the CCl_4_ solution eliminates the two sharp vibration peaks of free water at 3630 and 3710 cm^–1^ efficiently. As expected, removal of the dissolved water in the CCl_4_ solution via reaction with CaH_2_ noticeably reduces the number of interface-bonded water molecules on the QDs dispersed in the solution. Interestingly, the relatively broad peak at ∼3630 cm^–1^ remains strong, indicating that this peak is likely associated with one type of bonded water molecule at the inorganic–organic interface of the QDs.

CaH_2_ is insoluble in CCl_4_. Thus, it takes ∼10 minutes for CaH_2_ powder to reduce the number of water molecules at the interface per QD to a plateau (Fig. 4c). The plateau is approximately 40%–50% of that for the fresh CdSe QDs and ∼30% of that for the QDs equilibrated with water-saturated CCl_4_. Upon removal of CaH_2_ powder from the bottom of the solution, the number of interface-bonded water molecules per QD would quickly recover to a level similar to that of the fresh CdSe QDs in CCl_4_ (the point labeled as a cross in Fig. 4c), indicating the outstanding capability of water enrichment of the QDs with alkanoate ligands. Across all treatments associated with Fig. 4, the UV–Vis spectra of the QDs remain identical (Fig. 4d), indicating no influence on the inorganic core of the QDs.

### Bonding configuration of interface-bonded water molecules

**Figure 5.**
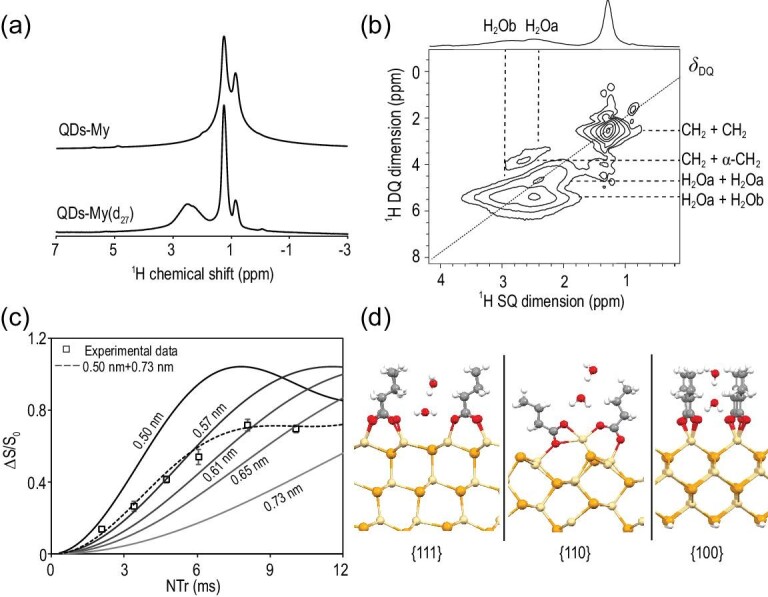
(a) Solid-state ^1^H MAS NMR spectra of CdSe QD-My and QD-My(d_27_). (b) DQ-SQ ^1^H–^1^H homonuclear two-dimensional correlation spectra of CdSe QD-My(d_27_). (c) REDOR curves (ΔS/S_0_) of the peak corresponding to water molecules and the calculated curves of different ^1^H-^113^Cd inter-nuclear distances. As labeled, the solid lines are calculated curves for specific single distances and the dashed line is the curve for two mixed distances. (d) Optimized structures of {111}, {110} and {100} facets with two water molecules in the supercell of the slab. The Monkhorst-Pack k-point sampling was generated with a 3 × 3 × 1 grid.

Typically, solid-state NMR signals of alkanoate ligands dominate the ^1^H spectrum. To minimize ^1^H signals of the aliphatic components, an ^1^H magic-angle spinning (MAS) spectrum (Fig. 5a) is collected using the CdSe QDs coated with 98% deuterated myristate ligands, denoted as QD-My(d_27_). Compared with the ^1^H signal of the QDs coated with regular (or protonated) myristate ligands (denoted as QD-My in Fig. 5a), the signals at 0.9 ppm (for CH_3_) and 1.2 ppm (for CH_2_) of QD-My(d_27_) (with 2% remaining protons) are significantly reduced and narrowed due to reduction of proton spin populations and their dipolar couplings. Evidently, a broad ^1^H peak emerges in the range between ∼1.5 and ∼3.5 ppm for the QD-My(d_27_) sample, which cannot be attributed to the myristate ligands and is consistent with the ^1^H of the interface-bonded water (see Fig. 2a). It was reported that the chemical shift of H_2_O in a non-polar environment can be dramatically smaller than that in an aqueous environment, which has often been observed in a number of nano-complex systems and surface structures [40–42].

Double quantum-single quantum (DQ-SQ) correlation experiments are performed on QD-My(d_27_) to probe different contributions to the emerged ^1^H peak through ^1^H–^1^H dipolar couplings. The DQ-SQ spectrum gives the double quantum frequency (δ_DQ_) along the y axis, which is the sum of single quantum chemical shifts (δ_SQ,1_ +δ_SQ,2_) for two coupled spin 1 and spin 2 (Fig. 5b). With the coupling information, we can distinguish different proton species and identify those in spatial proximity. The DQ dimension in Fig. 5b distinguishes four coupling schemes including δ_DQ_ = 2.4 ppm (1.2 + 1.2 ppm), δ_DQ_ = 4 ppm (1.2 + 2.8 ppm), δ_DQ_ = 4.8 ppm (2.4 + 2.4 ppm) and δ_DQ_ = 5.5 ppm (3.0 + 2.5 ppm). The δ_DQ_ = 2.4 ppm signal arises from the self-coupling of intramolecular CH_2_ groups (δ_SQ _= 1.2 ppm) on the alkyl chain. The δ_DQ_ = 4 ppm signal with low intensity is attributed to the α-CH_2_ (δ_SQ _= 2.8 ppm) of the myristate, which is coupled to intramolecular CH_2_ (δ_SQ _= 1.2 ppm). The δ_DQ_ = 4.8 and 5.5 ppm signals can be attributed to two different bonding environments of water species A and B with chemical shifts δ_SQ_ = 2.4 and 3 ppm, respectively. The δ_DQ_ = 4.8 ppm is the self-correlation water species A, and the δ_DQ_ = 5.5 ppm is the inter-correlation between water species A and B.

In summary, the solid-state NMR measurements above suggest that the interface-bonded water molecules are not in a homogeneous environment. Instead, there are at least two distinguishable locations. This conclusion is evidently in good accordance with the FTIR results. As shown above, the IR band of the interface-bonded water molecules is broad and shifts significantly in the low-frequency direction of those related to the free water molecules in the solvent.

### Distances between the interface-bonded water and the surface Cd sites

As summarized in Equation (4), exchange of the original alkanoate ligands by thiolate ones would largely eliminate the interface-bonded water molecules, suggesting that most of them are not directly attached to the inorganic surface. Solid-state NMR measurements can offer quantitative information on the ^1^H-^113^Cd inter-nuclear distance between the interface-bonded water and the Cd atoms on the nanocrystal surface. This is achieved by ^1^H{^113^Cd} rotational echo double resonance (REDOR) experiments on a doubly labeled QD(^113^Cd)-My(d_27_) sample (Fig. 5c) [43,44].

The isotope enrichment of ^113^Cd for CdSe QDs ensures all water molecules are coupled with their nearest Cd sites. The deuteration of myristate ligands occurs for the same reason mentioned above, i.e. to minimize the background ^1^H signals of the aliphatic chain. The REDOR experiments provide two signals. The S signal is obtained when both ^1^H and ^113^Cd resonances are irradiated, and the S_0_ reference signal is observed if only the ^1^H resonance is irradiated. The difference between S and S_0_, i.e ΔS, indicates the ^1^H signals that are coupled to ^113^Cd spins. The ratio of ΔS/S_0_ versus the periods of pulse irradiation can provide a quantitative measure of ^1^H–^113^Cd dipolar coupling (Figs S7 and S8, Supplementary Data). For rigid molecules, the dipolar coupling is directly related to the inter-nuclear distance (Fig. 5c). It is possible that the water molecules are rotating, which would lead to a certain level of uncertainty of distance. However, by reducing the measurement temperature of REDOR from 300 K to 196 K (Figs S7 and S8, Supplementary Data), we see little difference in the ΔS/S_0_ curve. This suggests the water molecules are rigid relative to the inorganic surface in the time scale of REDOR measurements, and therefore the inter-nuclear distance between ^1^H and ^113^Cd can be derived.

The REDOR measurements provide an overall response for the distance between the water molecules and the ^113^Cd spins at the inorganic–organic interface. Consistent with the other measurements discussed above, the experimental data cannot be fitted with single distance. Instead, the distance between water molecules and inorganic surface in the range of 0.5–0.7 nm can well fit the experimental data (Fig. 5c). This range means these water molecules are mostly not directly bonded to the surface sites of inorganic nanocrystals but are separated by a distance of roughly two or three chemical bonds (Table S1, Supplementary Data). To further uncover the bonding environment and facet-specific location of water molecules, we resort to detailed analysis with computational methods.

To model water adsorption at the interface of CdSe QDs, all three types of low-index facets of zinc-blende structure, namely {100}, {111} and {110}, are considered. As revealed in our recent study [27], alkanoate ligands are dominantly coordinated to the surface cadmium sites on the polar {100} and {111} facets of CdSe nanocrystals in chelating and bridging modes, respectively. In addition, neutral cadmium-carboxylate ligands form weak coordination on the non-polar {110} facets with a very low surface coverage. As shown in Fig. 5d, we use slab models with supercells to simulate ideal facets of CdSe nanocrystals and butyrate ligands to represent long-chain carboxylates to reduce the computational cost. On each kind of facet, one to three water molecules are considered in the supercell. The structures are optimized by density functional theory (DFT) with Quantum Espresso. The van der Waals density functional (vdW-DF) exchange-correlation function is adopted to describe electronic and hydrogen bond interactions. The plane-wave kinetic energy cutoff is set to be 60 Ry. The optimization is carried out until the forces on all atoms are lower than 0.025 eV/Å.

Figure 5d shows that, though coordination between alkanoate ligands and the inorganic surface differs from one type of facet to another, the water molecules at the interface are bonded to the hydrophilic region—specifically carboxylate groups of alkanoate ligands—in similar forms. Instead of direct bonding to the inorganic surface, the nearest monolayer of water molecules to the inorganic surface is hydrogen-bonded to two oxygen atoms from two adjacent carboxylate groups on the surface of a nanocrystal. The oxygen atom of the nearest water molecule is further connected to a hydrogen of another water molecule that forms the second monolayer of water molecules at the interface. The bonding energy of the hydrogen bonds is found to be quite high on all three types of facets (Table S2, Supplementary Data), consistent with their stability against vacuum treatments, thermal annealing and chemical drying procedures. The average distance between the hydrogen in the interface-bonded H_2_O and the surface cadmium sites on a nanocrystal is calculated to be in the range of 0.5 (the first monolayer) and 0.7 nm (the second monolayer), which fits quite well with the NMR results (Table S1, Supplementary Data).

### Interface-bonded water molecules in different types of inorganic nanocrystals with alkanoate ligands

**Figure 6.**
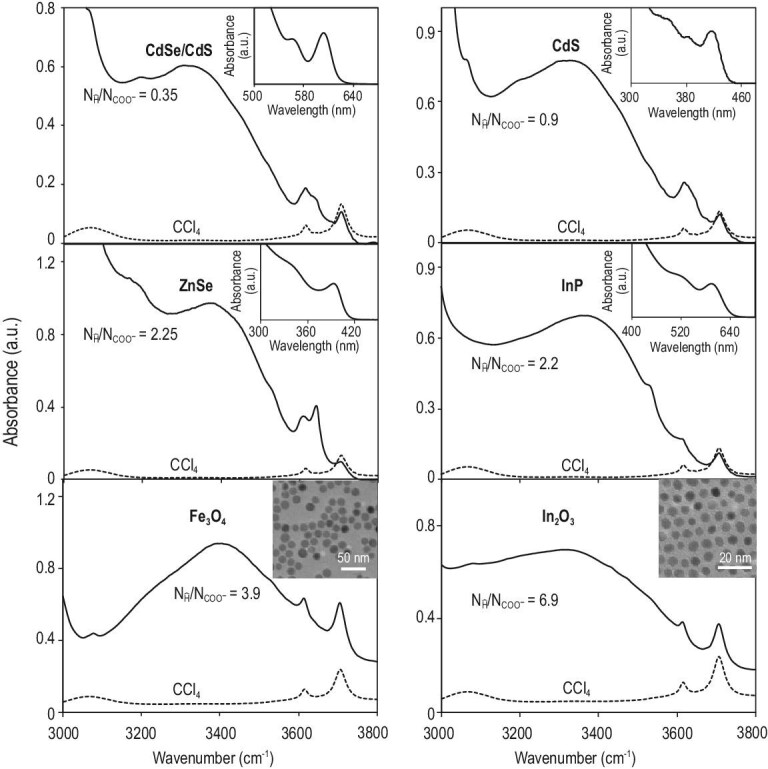
FTIR spectra of different types of inorganic nanocrystals coated with alkanoate ligands. FTIR spectrum of the solvent (CCl_4_) is provided as a reference. Insets: the corresponding UV–Vis spectra for QDs, and TEM images for oxide nanocrystals. The ratio between the active hydrogen atoms in the form of interface-bonded water molecules and carboxylate ligands is given in each plot.

The results above suggest that it is the hydrophilic region—specifically the carboxylate groups—of the alkanoate ligands that efficiently enrich water molecules at the inorganic–organic interface through hydrogen bonds between carboxylate groups and water molecules. Upon introduction of ‘greener synthetic routes’ for colloidal nanocrystals [12], alkanoate ligands have become the most applied ligands in the synthesis of nearly all types of high-quality inorganic nanocrystals in hydrocarbon solvents. Thus, interface-bonded water might be a common structure feature for high-quality colloidal nanocrystals. Results in Fig. 6 confirm the hypothesis. Different types of colloidal nanocrystals, including CdSe/CdS core/shell QDs [45], CdS QDs [46], ZnSe QDs [47], III-V QDs (InP) [48], Fe_3_O_4_ nanocrystals [13,32] and In_2_O_3_ nanocrystals [13,32], are synthesized in the most common hydrocarbon solvent (octadecene) and with metal alkanoates (sometimes with excess fatty acids) as both cationic precursors and ligands following standard methods. FTIR measurements reveal that, for all nanocrystals studied, the number of interface-bonded water molecules is approximately in the same order of magnitude as the number of alkanoate ligands.

### CONCLUSION AND PERSPECTIVE

In conclusion, within the inorganic-ligand interface of colloidal nanocrystals synthesized in non-aqueous solvents with alkanoate ligands, there are a large amount of bonded water molecules. These water molecules are enriched at the hydrophilic region of a colloidal nanocrystal, most of which are bonded to the carboxylate groups of the alkanoate ligands via hydrogen bonds. Results suggest that replacement of carboxylate groups through ligand exchange, such as replacing the alkanoate ligands with thiolate ligands, could dramatically decrease the number of interface-bonded water molecules. The common features of the inorganic–organic interface illustrated here open a new door not only for understanding optical, chemical, photo-chemical and photo-catalytic properties of high-quality colloidal nanocrystals, but also for designing synthetic and processing chemistry for these novel materials.

## Supplementary Material

nwab138_Supplemental_File
