# Supplementary material for: Water molecules bonded to the carboxylate groups at the inorganic–organic interface of an inorganic nanocrystal coated with alkanoate ligands
Source: Natl Sci Rev. 2021 Aug 4;9(2):nwab138. doi: 10.1093/nsr/nwab138 (PMC8882163; doi:10.1093/nsr/nwab138)
Supplement: nwab138_Supplemental_File [file nwab138_supplemental_file.pdf]

Supporting Information

Water monolayers at the interface between an inorganic nanocrystal and its alkanoate ligands

Jiongzhao Li,† Weicheng Cao,† Yufei Shu,† Haibing Zhang, Shudong Qian, Xueqian Kong,* Linjun Wang,* and Xiaogang Peng*

Zhejiang Key Laboratory of Excited-State Materials, Department of Chemistry, Zhejiang University, Hangzhou 310027, China

EXPERIMENTAL SECTION

Chemicals and Materials. Cadmium oxide (CdO, 99.99%), cadmium acetate dihydrate ($Cd(Ac)_2 \cdot 2H_2O$, 98+%), selenium powder (Se, 200 mesh, 99.999%), sulfur powder (S, 200 mesh, 99.999%), 1-octadecene (ODE, 90%), lauryl mercaptan (98+%) and oleic acid (90%) were purchased from Sigma-Aldrich. Tetrachloromethane (CCl_4, 99+%) was supplied by Aladdin Reagents. Indium acetate ($In(Ac)_3$, 99.99%), zinc nitrate hexahydrate ($Zn(NO_3)_2 \cdot 6H_2O$, 98%), n-dodecane (99%), tetramethylammonium hydroxide (98%), capric acid (HCa, 98%) and trioctylphosphine (TOP), Iron chloride ($FeCl_3 \cdot 6H_2O$, 98%), indium acetate ($In(Ac)_3$, 99.99%), 1-octadecyl alcohol (98%), myristic acid (98%), sodium hydroxide (NaOH, 98%) were purchased from Alfa Aesar. Stearic acid (HSt, 90+%) was supplied by Tokyo Chemical Industry. Chloroform-d ($CDCl_3$, 99.8%) was bought from J&K. Deuterium oxide (D_2O, 99.9%) was bought from Macklin. Cadmium oxide (^{113}Cd, 94.90%), myristic acid-d27 (d27, 98%) and methanol-d4 (CD_4O, 99.8%) was supplied by Cambridge Isotope Laboratories Inc. Tris(trimethylsilyl)phosphine ($(TMS)_3P$ in ODE solution (10wt%)) was purchased from Najing Tech Corporation. Ethyl acetate (AR), toluene (AR), acetonitrile (AR), hexane

(AR), methanol (AR), ethanol (AR) and hydrochloric acid (HCl, AR) were purchased form Sinopharm Reagents. All the chemicals and materials were used as received directly without further purification.

Synthesis of CdSe nanocrystals. The CdSe QDs were synthesized according to literature. [1] Se-suspension (Se-SUS) was prepared by dispersing Se powder (4 mmol) in ODE (10 mL). In a typical synthesis, CdO (0.8 mmol) and stearic acid (3.2 mmol) were loaded into a 50 mL three-neck flask with 24 mL of ODE. The mixture was heated to 280 °C to obtain a colorless solution. The temperature was promoted to 250 °C, and 1 mL of the Se-SUS was injected swiftly into the hot solution. The reaction was allowed to proceed at 250 °C. After 5 min, the Se-SUS was dropwise added into the solution with the speed of 0.2 mL/min until a targeted size of CdSe was obtained. Reaction process was monitored by taking aliquots for UV-vis measurements.

Synthesis of CdSe QDs for REDOR experiments share the same procedures as above with some changes in precursor materials. For instance, ^{113}CdO and myristic acid-d27 would replace CdO and stearic acid, respectively.

Synthesis of CdS nanocrystals. The CdS QDs were synthesized according to literature. [2] In a typical synthesis, CdO (0.8 mmol) and oleic acid (8 mmol) were loaded into a 50 mL three-neck flask with 24 mL of ODE. The mixture was heated to 200 °C to obtain a colorless solution. After the temperature was promoted to 250 °C, 1 mL of sulfur dissolved in ODE (0.4 M) was injected swiftly into the hot solution. The reaction was allowed to proceed at 250 °C for 10 min. Reaction process was monitored by taking aliquots for UV-vis measurements.

Synthesis of ZnSe nanocrystals. The ZnSe QDs are synthesized according to literature. [3] The synthetic procedures were divided into two steps. Firstly, oleic acid (10 mmol) was neutralized with an equal molar quantity of tetramethylammonium hydroxide in methanol (10 mL). Zinc nitrate hexahydrate (5 mmol) dispersed in 5 mL of methanol was mixed with this solution under vigorous stirring. White waxy zinc oleate precipitate was formed and collected by filtration and dried under vacuum overnight. Secondly, zinc oleate (1 mmol) in 10 mL of ODE were loaded into a 50 mL three-neck flask. The mixture was heated to 290 ºC and 1 mL Se-SUS (0.4 M) was injected swiftly into the hot solution. The reaction was allowed to proceed at this temperature for 15 min. Reaction process was monitored by taking aliquots for UV-vis measurements.

Synthesis of InP QDs. The InP QDs were synthesized according to literature. [4] Synthesis of InP QDs were divided into two steps. In the first step, indium stearate ($In(St)_3$) was prepared by heating 0.3 mmol of indium acetate and 0.9 mmol stearic acid at 150 ºC for 20 min. Argon was blowing continuously to remove acetic acid produced by the replacement reaction. Consequently, 0.4 mL (~0.9 mmol) of TOP was injected into the three-necked flask. Five minutes later, the mixture was allowed to cool down to 30 ºC by removing the heating mantle. Meanwhile, 0.48 mL of $(TMS)_3P$ in ODE solution (~0.15 mmol $(TMS)_3P$) was injected rapidly into the flask, and the reaction was allowed to proceed for approximately 1 minute and obtain the InP clusters solution.

In the second step, $In(St)_3$ (1 mmol) was prepared in the way described above in another flask. Next, 3 mL of ODE and 1.4 mL (~3 mmol) of TOP was added into the solution. After being kept at 150 ºC for 5 min, the mixture was heated to 260 ºC. InP clusters

solution prepared above was rapidly injected into the reaction mixture in the flask. After the first injection, the reaction temperature decreased to 240 °C and was allowed to remain at this temperature. For secondary-injection growth of InP QDs, additional InP clusters in solution was loaded into a syringe and dropped into the mixture at a speed of 0.9 mL/h, which was equivalent to the P precursor 0.135 mmol/h. UV-vis measurements of aliquots talking along the reaction were carried out to monitor the reaction. When a desired size of InP QDs was reached, the reaction mixture was allowed to cool down to room temperature by removing the heating mantle.

Synthesis of CdSe/CdS core/shell nanocrystals. The CdSe/CdS QDs were synthesized according to literature. [5] In a typical synthesis, $Cd(Ac)_2 \cdot 2H_2O$ (4 mmol), capric acid (4 mmol), oleic acid (12 mmol), purified CdSe core nanocrystals (0.4 μmol) with absorption peak at 550 nm and ODE (16 mL) were loaded in a 50 mL three-neck flask. The temperature was promoted to 260 °C and sulfur dissolved in ODE (0.2 M) was dropwise added into the solution with the speed of 4 mL/h until a targeted size of CdSe/CdS was obtained. Reaction process was monitored by taking aliquots for UV-vis measurements.

Synthesis of iron oxide nanocrystals. The iron oxide (magnetite, Fe_3O_4) nanocrystals was synthesized according to literature. [6] Firstly, the iron-oleate precursor was prepared by iron chloride reacting with NaOH and oleic acid. 1.35 g FeCl3・6H2O and 4.25 mL OA were dissolved in 25 mL methanol. After the solid dissolved, a NaOH solution with 0.6 g NaOH in 50 mL methanol was dropped under magnetic stirring conditions. The observed precipitate was washed by 100 mL methanol three times and dried under

vacuum at room temperature overnight. Then the dried precipitate was dissolved in 5 mL ODE at 80 oC and preserved as a stable stock solution. Next, 1 ml stock solution and 40 μL OA were dissolved in 4 mL ODE. The reaction mixture was heated to 300 oC under argon atmosphere and kept at that temperature for 30 min. The resulting solution containing the nanocrystals was then cooled to room temperature.

Purification of Iron Oxide Nanocrystals. The purification process for iron oxide nanocrystals consists of three steps. For the first step, the original reaction mixture with iron oxide nanocrystals was mixed with 12 mL of acetone. After vortex and centrifugation at 4000 rpm, the supernatant was removed. For the second step, 3 mL toluene was used to dissolve the precipitate, 3 mL of methanol was added and the mixture was centrifuged at 4000 rpm. The supernatant was removed. For the third step, the precipitate in step 2 was re-dissolved in 1 mL hexane and 10 mL acetonitrile was added. After vortex and centrifugation at 4000 rpm, the supernatant was removed. The remaining nanocrystals were dried under vacuum at room temperature for 30 minutes.

Synthesis of In_2O_3 nanocrystals. The In_2O_3 nanocrystals were synthesized according to a literature method. [7] Firstly, 0.3 mmol of $In(Ac)_3$ and 0.9 mmol myristic acid were mixed and heated at 150 ºC for 30 min. Argon was blowing continuously to remove the acetic acid produced during reaction. The product indium myristate was dissolved in 1 mL ODE and kept at 120 ºC. Next, 2.4345 g 1-octadecyl alcohol (9 mmol) and 18 mL ODE were loaded in a 50 mL three-necked flask and the mixture was heated to 290 ºC. 1 mL indium myristate in ODE was swiftly injected into the solution of 1-octadecyl

alcohol and ODE. The mixture was kept at 290 ºC for 30 min and then allowed to be cooled to room temperature.

Purification of nanocrystals. The purification process for CdSe nanocrystals reported in our previous literature consists of three steps. [8] For the first step, 2 mL of original reaction mixture with CdSe nanocrystals was mixed with 2 mL of ethyl acetate. After vortex and centrifugation at 4000 rpm, the supernatant was removed. Another 2 mL of ethyl acetate was added and repeated the above operation once again. For the second step, 1.1 mL of 10 vol% capric acid in toluene was used to dissolve the precipitate. The mixture was heated at 110 ºC until a clear solution was formed. Then, 1 mL of methanol was added and the mixture was centrifuged at 4000 rpm. The supernatant was removed, and the resulting precipitate was re-dissolved in 1.1 mL of 10 vol% capric acid in toluene to repeat this cycle once again. For the third step, the precipitate in step 2 was re-dissolved in 0.5 mL hexane and 8 mL acetonitrile was added. After vortex and centrifugation at 10000 rpm, the supernatant was removed, and the resulting precipitate was re-dissolved in 0.5 mL of hexane to repeat this cycle once again. The remaining nanocrystals were dried under vacuum at room temperature for 10 minutes. The same procedures were applied for purification of CdSe/CdS, CdS, ZnSe, and InP QDs but with no addition of capric acid in step 2.

Purification for iron oxide nanocrystals consists of three steps. For the first step, 3.6 mL of original reaction mixture with iron oxide nanocrystals was mixed with 14 mL of ethanol. After vortex and centrifugation at 4000 rpm, the supernatant was removed. For the second step, 4 mL toluene was used to dissolve the precipitate. Into the toluene solution, 4 mL of methanol was added and the mixture was centrifuged at 4000 rpm.

The supernatant was removed. For the third step, the precipitate in step 2 was re-dissolved in 1 mL hexane and 10 mL acetonitrile was added. After vortex and centrifugation at 4000 rpm, the supernatant was removed. The remaining nanocrystals were dried under vacuum at room temperature for 30 minutes.

The purification process for In_2O_3 nanocrystals consisted of three steps. For the first step, 19 mL of original reaction mixture with indium oxide nanocrystals was mixed with 20 mL of ethyl acetate. After vortex and centrifugation at 4000 rpm, the supernatant was removed. For the second step, 4mL toluene was used to dissolve the precipitate and the insoluble residue was removed by centrifugation. Into the clear toluene solution, 4 mL of acetone was added and the mixture was centrifuged at 4000 rpm. The supernatant was removed. For the third step, the precipitate in step 2 was re-dissolved in 1 mL hexane and 10 mL acetonitrile was added. After vortex and centrifugation at 4000 rpm, the supernatant was removed. The remaining nanocrystals were dried under vacuum at room temperature for 30 minutes.

Ligand exchange with thiol for CdSe QDs. In a typical procedure of ligand exchange, 0.4 mL of the purified QDs in n-dodecane (0.2 mM) were mixed with 0.2 mL of lauryl mercaptan (~ 0.8 mmol). Then the mixture was heated to 130 °C and kept at this temperature for 20 minutes. After the mixture was cooled down to room temperature, it was purified by the schemes below. Firstly, the QDs were precipitated by adding 2 mL of ethanol and centrifugation at 4000 rpm for 3 minutes. Secondly, the precipitate was washed by 2 mL of methanol twice. Finally, the precipitate was dried under vacuum at room temperature for 10 minutes. The loss of QDs during above process was determined by the procedures below. All supernatant in purification procedures were

collected. Ethanol and methanol in the supernatant were removed by rotary evaporation. The residual liquid was mixed with 2 mL of hexane and the sample was measured by UV-vis to determine the concentration of QDs. The purified QDs were dissolved in tetrachloromethane for FTIR measurements. Both the supernatant and the precipitate were acidized with aqua regia and diluted for AAS measurements if necessary.

Quantifying the number of carboxylates on QDs. In a typical procedure, 1 mL of purified QDs dissolved in n-dodecane (0.05 mM) was mixed with 1 mL of 6 M hydrochloric acid. The mixture was kept at 50 °C with stirring for 10 minutes. Then the fatty acid dissolved in n-dodecane was taken for FTIR measurements. The concentration of fatty acid was determined according to the standard curve of fatty acid in dodecane (Figure S3, Supporting Information). The concentration of QDs was determined according to the UV-vis using their extinction coefficient [8] prior to digestion.

Quantifying the number of thiolates on QDs. In a typical procedure, 2 mL of purified QDs in tetrachloromethane after ligand exchange with thiol (50 μM) was loaded in a quartz cell with 1 cm optical path for FTIR measurements. The concentration of thiolates was determined according to the standard curve of lauryl mercaptan in tetrachloromethane (Figure S4). The concentration of QDs was determined by the UV-vis.

Quantifying the amount of hydrogen in the form of interface-bonded water on QDs. In a typical procedure, 3 mL of purified QDs (0.4 mM) dissolved in tetrachloromethane was loaded into a 10 mL plastic centrifuge tube. Then 0.4 mL of water was added into the solution. The mixture was vibrated for 5 minutes at room

temperature to form a two-phase mixture. From the mixture, 2.5 mL of the tetrachloromethane phase was taken out carefully and mixed with 0.4 mL of deuterium oxide in another tube for 5 minutes to form another two-phase mixture. From this new mixture, 2 mL of the tetrachloromethane phase was taken out carefully and mixed with 0.2 mL water for 5 minutes. Finally, the water phase was taken for FTIR measurements. The concentration of deuterium oxide in water was determined according to the standard curve of deuterium oxide in water (Figure S6, Supporting Information). The concentration of QDs was determined according to the UV-vis.

Characterization and Measurements. UV−vis spectra were taken on Agilent Cary 4000 UV−vis spectrophotometer. FTIR spectra were recorded on a Nicolet 380 spectrometer. For FTIR measurements, different cuvettes were chosen according to different solvents. The CaF_2 cuvette with ~500 μm optical path was used for n-dodecane solutions. The CaF_2 cuvette with ~30 μm optical path was used for aqueous solutions. The quartz cuvette with 1 cm optical path was used for tetrachloromethane solutions. The atomic absorption measurements were taken on a PerkinElmer AA800 atomic absorption spectrometer. The solution state ^{1}H NMR spectra were taken on Agilent DD2-600 instrument. Solid-state NMR experiments were performed on a Bruker Avance III HD 400 MHz NMR spectrometer at resonance frequencies of 400.13 MHz for ^{1}H and 88.77 MHz for ^{113}Cd, using a 3.2 mm magic-angle spinning (MAS) probe. The spinning speed was 15 kHz. The ^{1}H signals were referenced to the methylene signal of adamantane at 1.8 ppm. For ^{1}H-^{113}Cd rotational-echo double resonance (REDOR) experiments at each dipolar recoupling time (Ntr), two signals were acquired with or without the ^{113}Cd irradiations which are labeled as S and S_0, respectively. For the 2D ^{1}H DQ-SQ correlation experiments, POST-C7 sequence was used for excitation and

reconversion of DQ coherences.

SI Figures

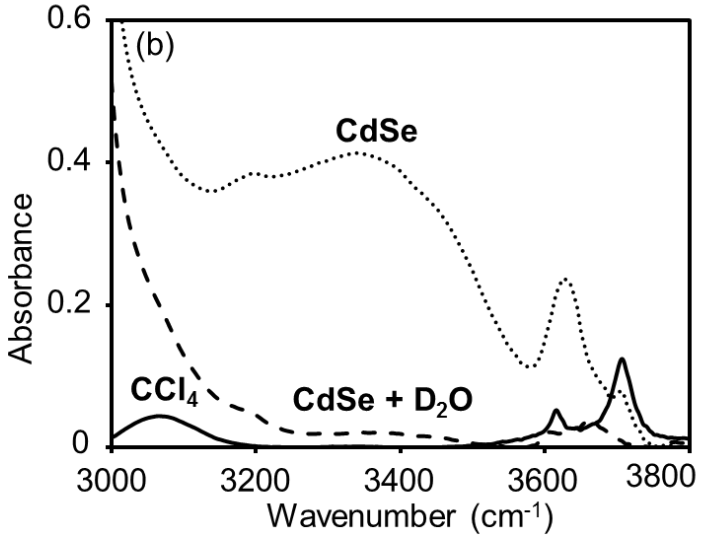

Figure S1. FTIR spectra between 3000 and 3800 cm^{-1} of CCl4 (black line), water-saturated CdSe QDs (black dot), exchanged CdSe QDs with deuteroxide (CdSe+D_2O, dotted line).

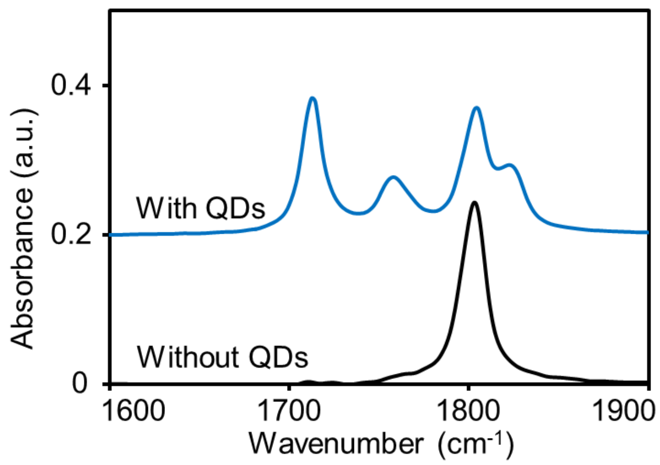

Figure S2. FTIR spectra of acryl chlorides in dodecane with and without QDs. For the case without QDs, the carbonyl vibration of acryl chlorides remains unchanged.

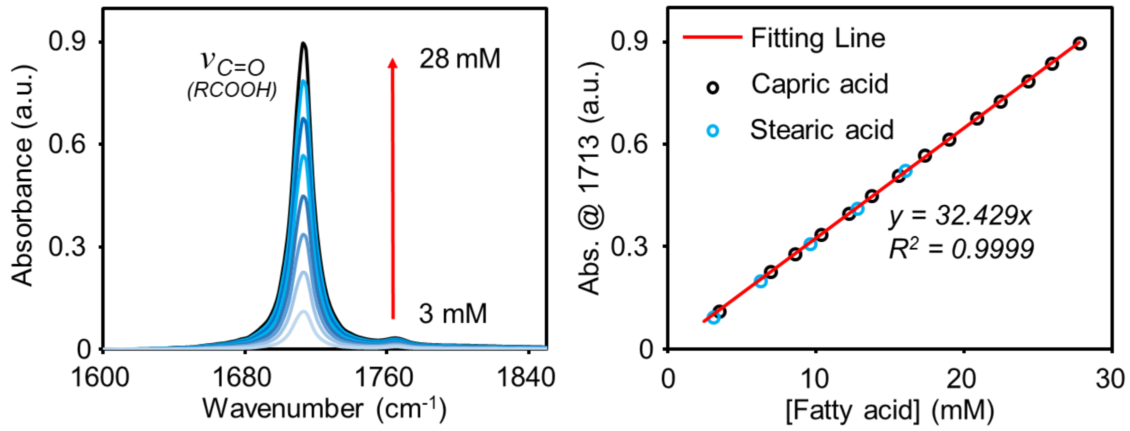

Figure S3. FTIR standard curve for carbonyl group of fatty acid. The solvent is dodecane and the cuvette is made of CaF_2 with optical path being 0.5 mm. Left graph is the experimental FTIR spectra. Linear fitting line is plotted by absorbance at 1713 cm^{-1} verse concentration of fatty acids (right). Two types of fatty acids follow the same linear function.

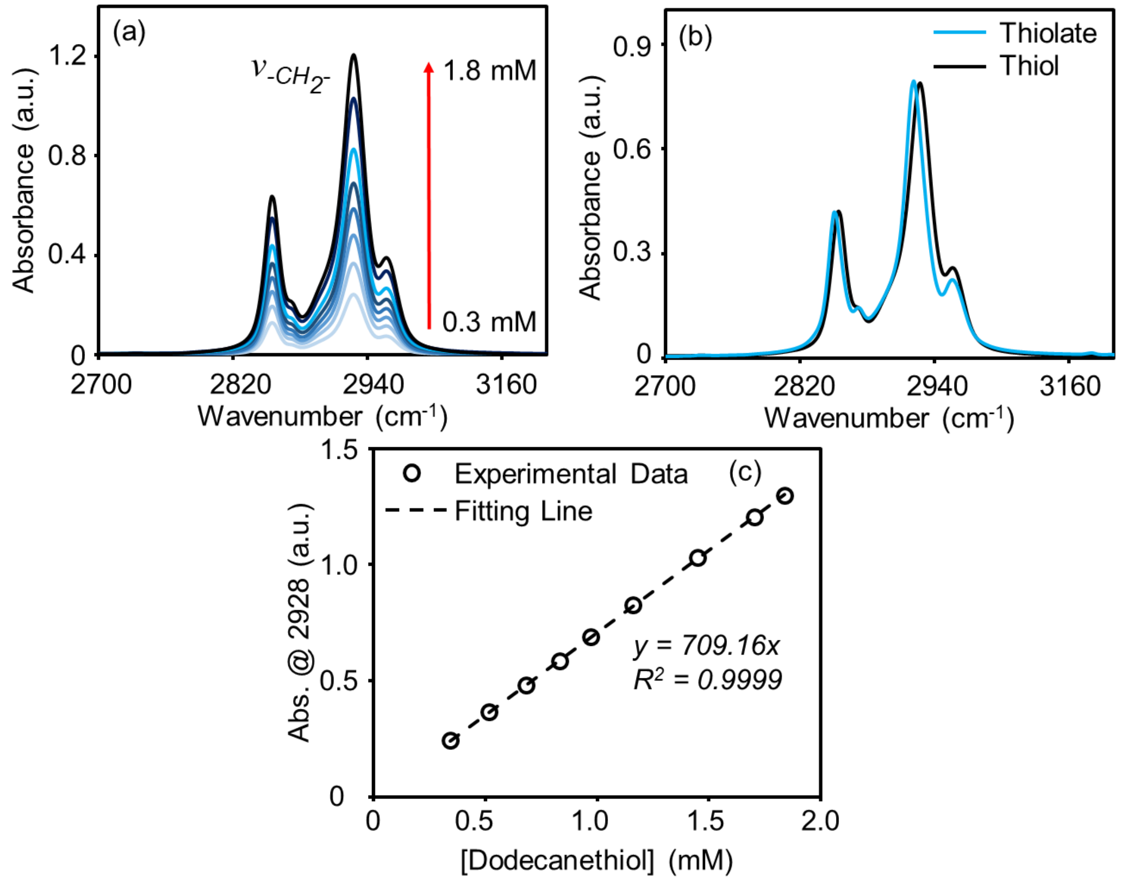

Figure S4. FTIR standard curve for hydrocarbon in dodecanethiol, which is the thiol molecules applied for ligand exchange related to Figure 2. The solvent is tetrachloromethane and the cuvette is made of quartz with optical path being 1 cm. (a) the experimental FTIR spectra. (b) FRIT spectra of thiol and thiolates with the same concentration. (c) Linear fitting line is plotted by absorbance at 2923 cm^{-1} verse concentration of dodecanethiol.

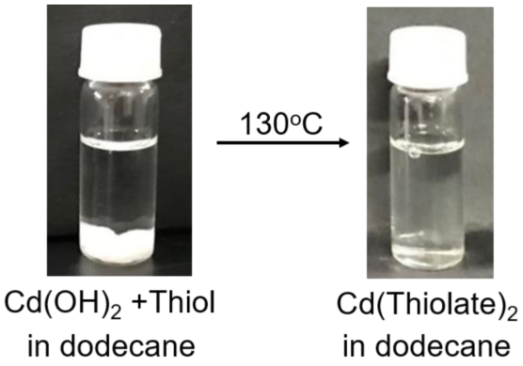

Figure S5. Visual illustration of raction between $Cd(OH)_2$ and dodecanethiol in dodecane at 130°C.

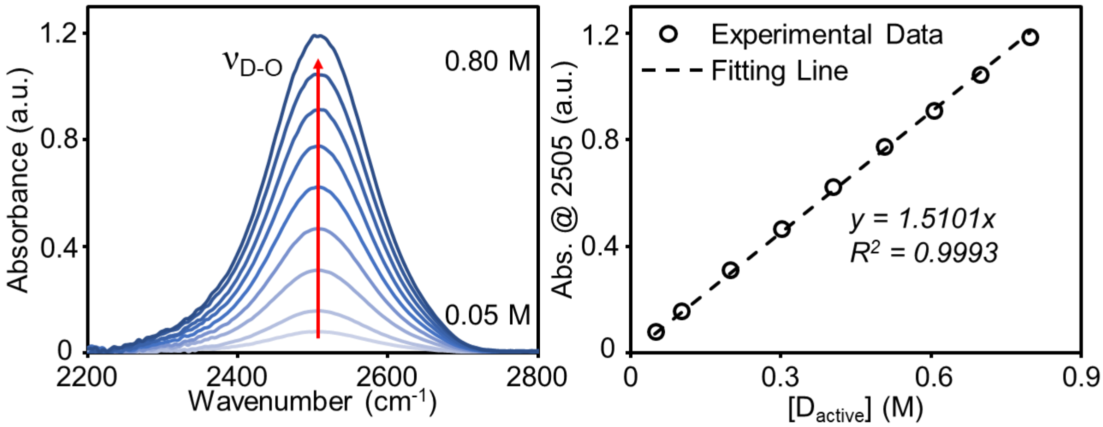

Figure S6. Left: experimental FTIR spectra of D_2O in H_2O with different concentrations in the CaF_2 cuvette with optical path being ~30 μm. Right: absorbance at 2505 cm^{-1} verses concentration of deuterium oxide (counted as deuterium, D_{active}).

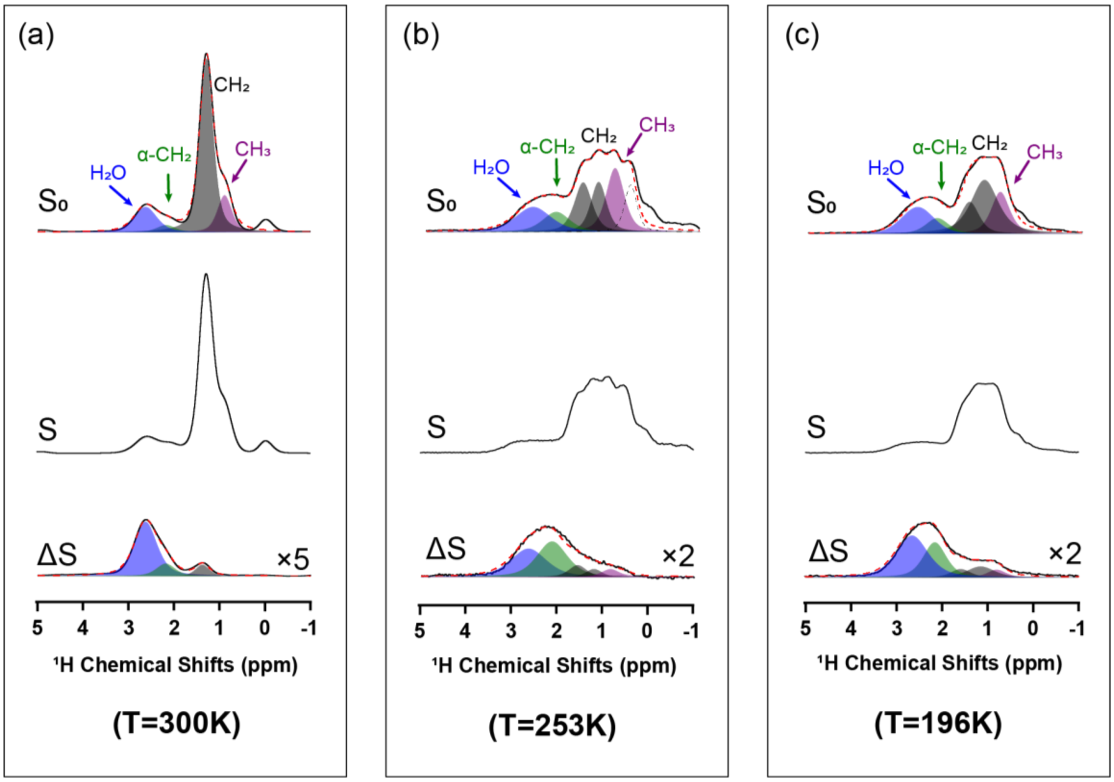

Figure S7. The REDOR ^{1}H-^{113}Cd NMR spectrums of CdSe QDs-My(d27) and at (a) 300K, (b) 253K and (c) 196K: the full echo spectra (S_0, top), the recoupling spectra (S, middle) and their difference spectra (ΔS, bottom). Their dephasing time were ~10 ms. The ^{1}H spectrums can be deconvoluted into several components, including H_2O(~2.6 ppm, Blue), the α-CH_2(~2.1 ppm, Green), CH_2(1.2-1.5 ppm, Black) and CH_3(~0.85 ppm, Purple) groups of the myristate ligands.

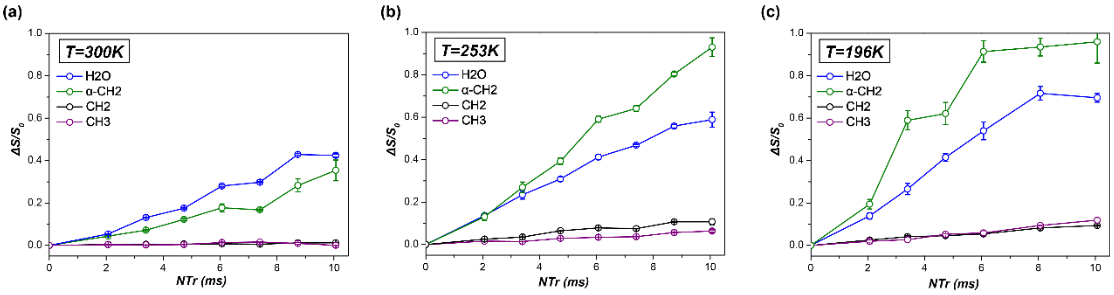

Figure S8. The ^{1}H-^{113}Cd REDOR dephasing curves for H_2O (Blue circles), α-CH_2 (Green circles), CH_2 (Black circles) and CH_3 (Purple circles) of CdSe QDs-My(d27) at (a) 300K, (b) 253K and (c) 196K.

Computational Details and Discussion

To model water adsorption on the surface of CdSe quantum dots, we considered three types of low-index facets, namely, {100}, {111}, and {110}. As revealed in our recent study,[9] chelating and bridging modes are dominant coordinations of carboxylate ligands on the polar {100} and {111} facets of CdSe nanocrystals, while cadmium carboxylates form weak coordinations on the nonpolar {110} facets. As shown in Figure S9, we use slab models with supercells to simulate ideal facets of CdSe nanocrystals and represent long-chain carboxylates as butyrate ligands to reduce the computational cost. On each kind of facet, one to three water molecules are considered in the supercell (see Figures S10-S12). The structures were optimized by the density functional theory with Quantum Espresso.[10] The vdW-DF[11] exchange-correlation functional was adopted to describe electronic and hydrogen-bond interactions. The plane-wave kinetic energy cutoff was set to be 60 Ry. The optimizations were completed until forces on all atoms were lower than 0.025 eV/ Å.

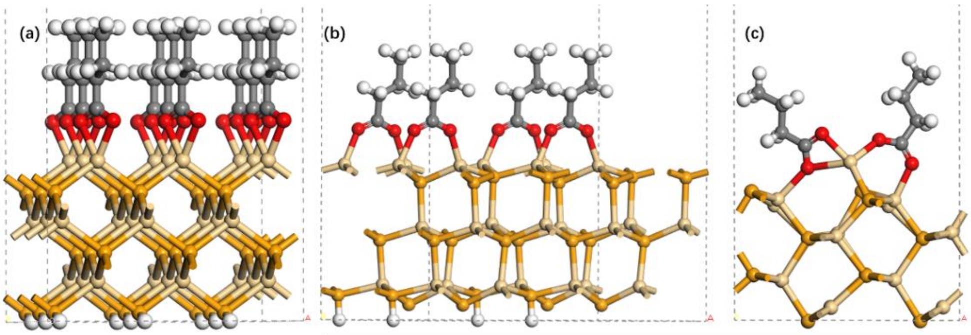

Figure S9. Optimized structures of (a) {100}, (b) {111}, and (c) {110} facets of CdSe nanocrystals.

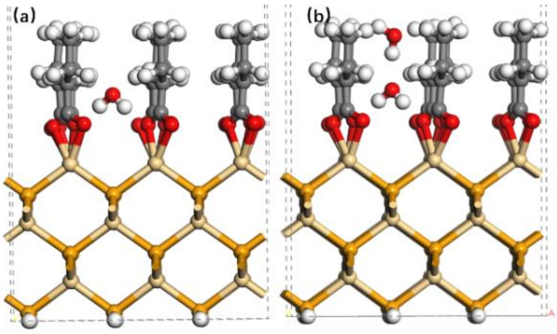

Figure S10. Optimized structures of {100} facets with (a) one and (b) two water molecules in the supercell of the slab. The Monkhorst-Pack k-point sampling was generated with a 3×3×1 grid.

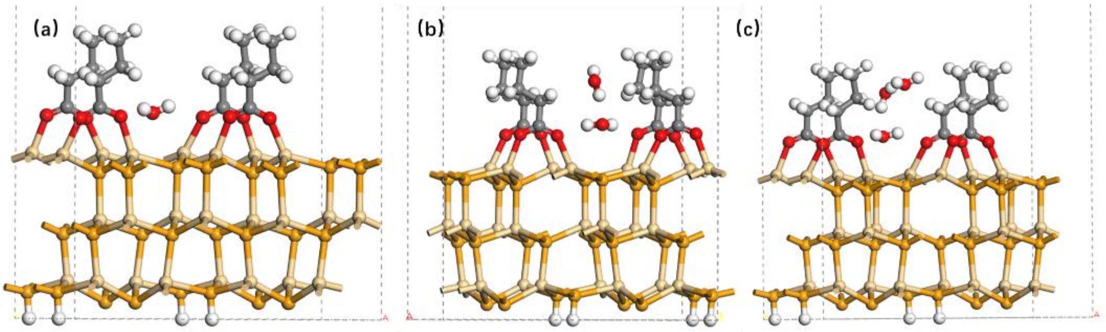

Figure S11. Optimized structures of {111} facets with (a) one, (b) two, and (c) three water molecules in the supercell of the slab. The Monkhorst-Pack k-point sampling was generated with a 3×5×1 grid.

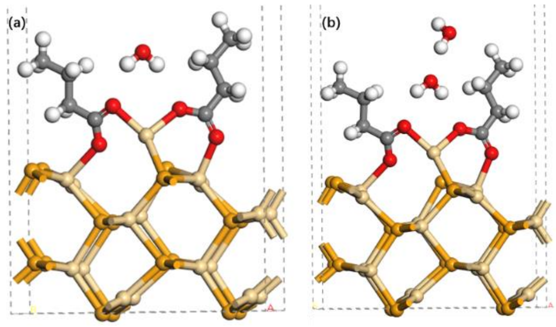

Figure S12. Optimized structures of {110} facets with (a) one and (b) two water molecules in the supercell of the slab. The Monkhorst-Pack k-point sampling was generated with a 5×5×1 grid.

	Average distance between H and the closest Cd/Å			
	{100}	{111}	{110}	Average
H_2O-1	4.02	3.97	5.57	4.52
H_2O-2	6.75	6.01	8.49	7.08

Table S1. Average distance between a hydrogen atom in the water molecules and the closest cadmium atom on different facets of the CdSe nanocrystals.

We further calculated the binding energies of water molecules on the different facets. For the *N*th water molecule adsorbed on a surface of CdSe nanocrystals, the binding energy (ΔE_N) is calculated by

$$\Delta E_N = E_N - E_{N-1} - E_{H_2O}, \quad (1)$$

where E_N is the energy of the slab with N adsorbed water molecules, $E_{N\text{-}1}$ is the energy of the slab with N-1 adsorbed water molecules, and E_{H_2O} is the energy of an isolated water molecule. Thereby, E_0 denotes the energy of the slab without water molecules (see Figure S9). As shown in Table S2, the binding energies of water molecules all fall in the range of -5 ~ -15 kcal/mol, which are typical values for intermolecular hydrogen bonds. For {100} and {110} facets, the second water molecule binds slightly stronger than the first water molecule due to the higher flexibility for geometry relaxation. In

contrast, the binding of water molecules on the {111} facet gets weaker with the adsorption of more water molecules. This is also reasonable because the bridging mode is associated with a lower ligand density and more hydrogen bonds are present between the first layer of water molecules and the carboxylate groups of surface ligands. Due to the higher binding energies of water molecules on the {111} facets, water adsorption seems to favor the {111} facets than the other two facets.

	{100}	{110}	{111}
E_0 (Ry)	-5062.175	-2695.626	-3996.691
E_1 (Ry)	-5098.564	-2732.007	-4033.097
E_2 (Ry)	-5134.958	-2768.393	-4069.490
E_3 (Ry)	\	\	-4105.877
ΔE_1 (kcal/mol)	-7.78	-5.27	-13.11
ΔE_2 (kcal/mol)	-9.35	-6.84	-9.04
ΔE_3 (kcal/mol)	\	\	-7.15

Table S2. Energies of the optimized structures, and binding energies of water molecules on {100}, {110}, {111} facets. The energy of an isolated water molecule is -36.3642 Ry.

References

1. Pu, C.; Zhou, J.; Lai, R.; Niu, Y.; Nan, W.; Peng, X., Highly reactive, flexible yet green Se precursor for metal selenide nanocrystals: Se-octadecene suspension (Se-SUS). *Nano Res.* **2013,** *6* (9), 652-670.
2. Yu, W. W.; Peng, X., Formation of High-Quality CdS and Other II–VI Semiconductor Nanocrystals in Noncoordinating Solvents: Tunable Reactivity of Monomers. *Angew. Chem., Int. Ed.* **2002,** *41* (13), 2368-2371.
3. Lin, S.; Li, J.; Pu, C.; Lei, H.; Zhu, M.; Qin, H.; Peng, X., Surface and intrinsic contributions to extinction properties of ZnSe quantum dots. *Nano Res.* **2020,** *13* (3), 824-831.
4. Xu, Z.; Li, Y.; Li, J.; Pu, C.; Zhou, J.; Lv, L.; Peng, X., Formation of Size-Tunable and Nearly Monodisperse InP Nanocrystals: Chemical Reactions and Controlled Synthesis. *Chem. Mater.* **2019,** *31* (14), 5331-5341.
5. Zhou, J.; Pu, C.; Jiao, T.; Hou, X.; Peng, X., A Two-Step Synthetic Strategy toward Monodisperse Colloidal CdSe and CdSe/CdS Core/Shell Nanocrystals. *J. Am. Chem. Soc.* **2016,** *138* (20), 6475-6483.
6. Jana, N. R.; Chen, Y.; Peng, X., Size- and Shape-Controlled Magnetic (Cr, Mn, Fe, Co, Ni) Oxide Nanocrystals via a Simple and General Approach. *Chem. Mater.* **2004,** *16* (20), 3931-3935.

7. Narayanaswamy, A.; Xu, H.; Pradhan, N.; Kim, M.; Peng, X., Formation of Nearly Monodisperse In2O3 Nanodots and Oriented-Attached Nanoflowers: Hydrolysis and Alcoholysis vs Pyrolysis. *J. Am. Chem. Soc.* **2006,** *128* (31), 10310-10319.
8. Li, J.; Chen, J.; Shen, Y.; Peng, X., Extinction coefficient per CdE (E = Se or S) unit for zinc-blende CdE nanocrystals. *Nano Res.* **2018,** *11* (8), 3991-4004.
9. Zhang, J.; Zhang, H.; Cao, W.; Pang, Z.; Li, J.; Shu, Y.; Zhu, C.; Kong, X.; Wang, L.; Peng, X., Identification of Facet-Dependent Coordination Structures of Carboxylate Ligands on CdSe Nanocrystals. *J. Am. Chem. Soc.* **2019,** *141* (39), 15675-15683.
10. Giannozzi, P.; Baroni, S.; Bonini, N.; Calandra, M.; Car, R.; Cavazzoni, C.; Ceresoli, D.; Chiarotti, G. L.; Cococcioni, M.; Dabo, I.; Dal Corso, A.; de Gironcoli, S.; Fabris, S.; Fratesi, G.; Gebauer, R.; Gerstmann, U.; Gougoussis, C.; Kokalj, A.; Lazzeri, M.; Martin-Samos, L.; Marzari, N.; Mauri, F.; Mazzarello, R.; Paolini, S.; Pasquarello, A.; Paulatto, L.; Sbraccia, C.; Scandolo, S.; Sclauzero, G.; Seitsonen, A. P.; Smogunov, A.; Umari, P.; Wentzcovitch, R. M., QUANTUM ESPRESSO: a modular and open-source software project for quantum simulations of materials. *Journal of Physics: Condensed Matter* **2009,** *21* (39), 395502.
11. Dion, M.; Rydberg, H.; Schröder, E.; Langreth, D. C.; Lundqvist, B. I., Van der Waals Density Functional for General Geometries. *Physical Review Letters* **2004,** *92* (24), 246401.
